# The resident experience with psychological safety during interprofessional critical event debriefings

**DOI:** 10.1002/aet2.10864

**Published:** 2023-04-01

**Authors:** Lily Hitchner, Mackensie Yore, Charney Burk, Jessica Mason, Stacy Sawtelle Vohra

**Affiliations:** ^1^ Department of Emergency Medicine UCSF Fresno Medical Education Program Fresno California USA; ^2^ UCLA National Clinician Scholars Program, Department of Emergency Medicine Greater Los Angeles VA Medical Center California Los Angeles USA; ^3^ Department of Emergency Medicine John Peter Smith Hospital Fort Worth Texas USA

**Keywords:** critical event debriefing, interprofessional feedback, psychological safety

## Abstract

**Objectives:**

Interprofessional feedback and teamwork skills training are important in graduate medical education. Critical event debriefing is a unique interprofessional team training opportunity in the emergency department. While potentially educational, these varied, high‐stakes events can threaten psychological safety for learners. This is a qualitative study of emergency medicine resident physicians’ experience of interprofessional feedback during critical event debriefing to characterize factors that impact their psychological safety.

**Methods:**

The authors conduced semistructured interviews with resident physicians who were the physician team leader during a critical event debriefing. Interviews were coded and themes were generated using a general inductive approach and concepts from social ecological theory.

**Results:**

Eight residents were interviewed. The findings suggest that cultivating a safe learning environment for residents during debriefings involves the following: (1) allowing space for validating statements, (2) supporting strong interprofessional relationships, (3) providing structured opportunities for interprofessional learning, (4) encouraging attendings to model vulnerability, (5) standardizing the process of debriefing, (6) rejecting unprofessional behavior, and (7) creating the time and space for the process in the workplace.

**Conclusions:**

Given the numerous intrapersonal, interpersonal, and institutional factors at play, educators should be sensitive to times when a resident cannot engage due to unaddressed threats to their psychological safety. Educators can address these threats in real time and over the course of a resident's training to enhance psychological safety and the potential educational impact of critical event debriefing.

## INTRODUCTION

### Background

Interprofessional feedback and team skills training are important in graduate medical education and required by the Accreditation Council for Graduate Medical Education (ACGME).^1^ Critical event debriefing is the practice of standardized team reflection aimed at incorporating improved behaviors and teamwork skills into clinical practice. These sessions are unique opportunities to provide direct interprofessional feedback to the resident physician in conjunction with the shared learning of debriefing.^2^, ^3^ Feedback and debriefing, both experience‐informed dialogues or “learning conversations,” have common goals and attributes but different theoretical roots in medical education literature; critical event debriefing challenges the “contextual divide between feedback and debriefing, highlighting the overlap in purpose and structure.”^4^ Integrating feedback and debriefing into one standardized interprofessional session may not only be practical but could advance both conversational strategies as educational tools.

Much of the literature on interprofessional feedback after critical events has been conducted in simulated sessions, which differ from critical event debriefings in important ways. Simulated sessions have been coined “safe containers” for learning, with a predictable structure, and trained facilitators.^5^, ^6^ Unlike simulated patient care encounters, critical events in the clinical setting are high stakes, unpredictable, and without protected time and space to debrief. Furthermore, critical event debriefing participants—both the givers and the recipients of feedback—have varied relationships and levels of training. Because the clinical team composition is often different from one critical event to the next, interprofessional feedback in critical event debriefings may not have the advantage of an established teacher–learner relationship or educational alliance often present in simulations.^7^

It is important to understand the residents’ sense of psychological safety during clinical critical event debriefing and feedback sessions to determine if, and when, educators can capitalize on these learning conversations. Dr. Amy Edmonson defines team psychological safety as “a shared belief that the team is safe for interpersonal risk taking.”^8^ In critical event debriefings, team members may take risks by admitting errors or discussing opportunities for individual and team growth in clinical care, procedural skills, communication, or leadership skills. These real‐world learning conversations in a team setting benefit from mutual respect, trust in the team, caring for each other as individuals, and confidence in oneself. When learners feel unrestrained from the judgment of the team and the feeling that they need to always project competence, they can fully engage with the learning opportunities, are more productive, and are more satisfied with their learning environment.^6^, ^7^, ^9^, ^10^, ^11^ Alternatively, psychological distress leads to poor workplace relationships, provider burnout, and cognitive barriers to learning.^6^, ^10^

### Goals of this investigation

When residents engage in critical event debriefings, they may experience greater benefits from the learning conversation if they have greater psychological safety. Educators should be sensitive to times when a learner experiences threats to their psychological safety and address these threats. This study aims to explore residents’ experiences with psychological safety during debriefings to identify the success factors and limitations in these dynamic and complex learning conversations.

## METHODS

### Study design and setting

This was a qualitative study examining emergency medicine resident physicians’ experiences with psychological safety while receiving interprofessional feedback in an established critical event debriefing program. The study was conducted at the University of California San Francisco–Fresno (UCSF Fresno) Department of Emergency Medicine at Community Regional Medical Center (CRMC). CRMC is a Level I trauma and burn center located in Fresno, California, with an emergency department (ED) volume of approximately 110,000 annually. CRMC serves as the main site for the UCSF Fresno Department of Emergency Medicine residency program with 44 residents spread over 4 class years. The critical event debriefing program was started in winter 2017 as a joint effort between CRMC and UCSF Fresno Department of Emergency Medicine. In addition to the emergency medicine resident physician team leader, these debriefings can include emergency medical system (EMS) personnel, nurses, technicians, respiratory therapists, social workers, pharmacists, attending physicians, other residents or medical students, and occasionally consultants. They usually occur immediately after a critical event (i.e., cardiac arrest, difficult intubation, unexpected patient decline, precipitous delivery, rare ED procedure, medication or communication error). Anyone on the team can initiate a debriefing; they are optional and are led by the resident physician team leader. During the debriefing, the team reviews group performance and is prompted to provide specific feedback to the resident physician team leader. A standardized critical event debriefing form is used to guide the session (Appendix B). Each session lasts approximately 5 to 15 min. The process and data collection form were adapted from Dr. Paul Mullan's DISCERN program.^12^ These forms are collected and reviewed by the ED medical director and an assistant residency program director to address both systems and educational issues, respectively.

### Selection of participants

All resident physician team leaders in a critical event debriefing in the previous 3 months at the start of the study period were invited to participate via email.

### Measurements

One researcher, LH, conducted semistructured interviews asking residents to (1) describe their experiences participating in the debriefing sessions over the course of their residency, (2) discuss the nature of feedback received, and (3) reflect on factors that made them feel safe or less safe during these sessions (Appendix A). Interviews were conducted via Zoom video conferencing and transcribed prior to analysis. Interviews lasted approximately 30 min each and no one else was present during the interviews. All interview transcripts were deidentified to maintain anonymity and reduce bias. This project was approved by the Community Medical Centers Institutional Review Board in Fresno, California.

### Data analysis

We coded interview transcripts and generated themes with a general inductive approach using concepts from social ecological theory. Social ecological theory views individual behavior as a complex interplay between intrapersonal factors, interpersonal processes, institutional factors, community factors, and public policy.^12^ The social ecological model for health promotion helps us understand that changes in the social environment produce changes in individuals, and those individual changes then alter the environment and culture of an institution.^12^ For the purposes of this analysis, we focused on the first three components to analyze individual narratives in this research setting. This organizational framework, referred to as a transactional model between the individual and the environment, shaped how we categorized and understood the limiting and success factors of the learner's psychological safety in clinical critical event debriefing sessions.

Two researchers (LH, SSV) coded the data separately using NVivo software, a program to aid in the processing and analysis of qualitative data. They met regularly to develop and revise the coding key, review dominant themes, define and name the themes, and discuss interpretation of the data and conclusions.^13^ A general inductive approach was used, and the final themes were discussed and approved by all investigators.

The researchers practiced reflexivity to consider how their position and participation in debriefings could influence data collection. During the interviews, the interviewer (LH) acknowledged her role as a champion of the critical event debriefing program, a resident evaluator, and an attending physician. The researcher acknowledged this in the interviews to address potential power dynamics by emphasizing the goal of understanding the process and improving the feedback process to the study participants. CB, LH, and MY received feedback as residents during debriefing sessions and acknowledged their own personal experiences when analyzing the data. JM, LH, and SSV acknowledged their role as attending physicians participating in debriefing sessions and champions of the program.

## RESULTS

### Characteristics of study subjects

Fifteen emergency medicine residents led a critical event debriefing in the previous 3 months and were invited to participate in the study via email. Eight residents responded to the invitation to enroll and all eight completed an interview. This sample included one second‐year, three third‐year, and four fourth‐year residents; five were female and three were male. Residents were not queried as to why they did or did not respond to the invitation to participate in the study.

### Main results

Ten major themes were identified and grouped into (1) intrapersonal, (2) interpersonal, and (3) institutional factors that impacted resident psychological safety during a critical event debriefing. Table 1 provides an overview of the major themes with representative quotes.

**TABLE 1:** Summary of key themes with representative quotes.

Topic Area	Theme	Representative quotes
Intrapersonal	Validation from the interprofessional team during debriefing contributes to professional identity development.	“I think it is good having other people share the journey, be present, maybe make you feel just a little bit more confident that despite something not going as you had hoped, there were things that were good, and you know these things.” (int 1)
		“I came across as very nervous when I was early in second year, so I got a bunch of pep talks in my early debriefings, people reassuring me that I was doing a good job or that I was being loud enough for running the room adequately.” (int 5)
	Prior experience with feedback increased comfort with team debriefing.	“I think when you play sports, you know your coaches are always giving you feedback all the time and so you just kind of get used to like feeling vulnerable. But you kind of realize it's not personal or you just realize, it's just part of it, you know.” (int 8)
		“If I'm not getting feedback, then I'm not going to be improving. And then I'm going to be doing wrong by somebody in the future because I didn't receive feedback and I'm going to learn a habit that is bad … I don't want to just slide by and be okay, I want to be a good physician.” (int 6)
	Residents may prioritize self‐preservation over debriefings.	“When you are transitioning to a higher acuity zone there is a potential for bad things to happen. I think it is a little bit harder to be as vulnerable … sometimes the feedback that you're getting from many different sources can be a little bit overwhelming, it's all important, but it can be just a little bit hard to chew.” (int 1)
		“I just always kind of baseline feel like I'm doing a bad job … I just don't want to like open up to receive negative feedback in front of other people because I feel like that's what I'm going to receive if I ask for it.” (int 5)
		“… actually, that's the only time that I have bawled afterwards. [Name omitted] gave me a hug and it was not a, I think, I would not have been in an emotionally good place to take that feedback at that time.” (int 7)
Interpersonal	Residents consider preexisting relationships and respect for interprofessional team members prior to initiating debriefing.	“I think with time as you build up more confidence and especially when you build up relationships with your team, they are able to be more honest with you and I think when you take their feedback, you take it very honestly also. You know that they're not doing it because they don't trust you or believe in you, they're doing it because they want to see you grow and I think that just takes time.” (int 2)
		“Sometimes for example when you go into [the trauma zone] and you're new … it takes a little bit of time for you to develop those relationships with the trauma nurses. So, when you're in the debrief you almost kind of want to maintain a calm, cool, collected attitude a little bit.” (int 6)
		“I feel just like attendings, the nurses watch us grow and they know what our weak points and strong points are at least the ones that have been up in [the high acuity zones] for a long time and so they have kind of watched our progression over years and they have a little bit better insight into what we're doing well and not doing well and so they can be really valuable people to get feedback from.” (int 5)
	Attending partnership matters.	“There have been like a couple situations where like the attending perhaps I was less comfortable with or who I felt was like not as open to that sort of thing where I kind of just wanted to focus on more you know the positives and negatives of the case rather than individual performance, particularly my own performance.” (int 5)
		“An attending sets the tone for what type of feedback is being given. Like if the attending starts with a canned response like people are probably going to give canned responses and if the attending gives like a very specific useful response, people are going to probably continue that because they're setting the tone.” (int 4)
	Unprofessional behavior limits safety.	“I think when it gets emotional and volatile and when people start either yelling, cussing, or being more abrasive … that makes me less inclined to want to engage with that person.” (int 8)
	The resident's perception of their position on the team affects their attitudes toward debriefing.	“I think once you realize that can actually contribute to the way that you are as provider and a person then maybe it motivates you to do more. That the end of a code is not the end of that experience. There could be something you can do to motivate yourself and the team.” (int 2)
		“I think that for me feedback is a way to give a lot of meaning and purpose to really tough situations. Like okay, this is really hard, this patient is really sick, like what can we learn from this to make this situation, as hard and tough and it was, or if someone passed, you know, how sad it was for the family … how do I make this experience more meaningful so that person and that person's illness has a bigger purpose than even just that experience?” (int 6)
Institutional	A standardized process lowers barriers to initiating team debriefing.	“I'm certainly not a person to push people if I get an immediate like reluctance to it. I think initially there was that and it's become that debriefing has become a little bit more ingrained in the culture where it becomes easier, and because it becomes easier, I feel safer even to bring it up in the first place if that makes sense?” (int 4)
		“It's understood that it's to improve patient care and it improves the way that the team can provide care for them, for the patient, and so I think the feedback that's delivered, it's for good intentions. If anything, the feedback that's received is ultimately to become a better clinician from the resident standpoint and from our team's standpoint it's just to be better providers. So, I think it's a good setting for us to be honest with each other and to kind of point out some issues that we can hope to improve on in the future.” (int 2)
	Lack of private space and uninterrupted time limits engagement.	“I mean our department is kind of hectic all the time and it's hard to get people to all gather in one space at all but then to find a space where you can kind of talk and have things be quiet and subtle for a little while is difficult.” (int 5)
		“I've always done the debrief in the room with the patient which I think actually could interfere especially if the patient has passed and such.” (int 6)
	Having a department with multiple interprofessional learners increases resident's participation.	“I think another thing that kind of made me feel very safe is the fact that the nurses that were there that day were also learners … and so those people also had their own feedback for themselves and so I felt like everyone was kind of on the same page of like okay we're all trying to figure this out together, and we all kind of know how things are supposed to happen, but someone is better at this than us.” (int 3)
		“I think when you're willing to be vulnerable you like drop yourself way down [in the hierarchy] and you allow yourself to be a position where if people want to, they could really take advantage of that. But I think in my experience when people see you do that, they also are willing to kind of step down into your level and open up the space where you all are at that and improving and growing.” (int 6)

#### Intrapersonal factors

Residents found meaning in validating statements from the interprofessional team. This contributed to their confidence in their roles as physician team leaders during resuscitations, in the medical care they provided in cases of poor patient outcomes, and in their medical knowledge at their stage of training. Having their actions validated by the team helped counter negative internal dialogue. Residents appreciated hearing that a poor outcome was unavoidable or that similar decisions were made by a different set of providers facing a similar clinical scenario. Validation during the current debriefing session and in prior sessions increased their willingness to ask members of the interprofessional team to share their perspective about the case and about their performance as team leader.

Residents cited prior experience with feedback, both within and outside of medicine, and a positive mindset around feedback as increasing their comfort in the debriefing sessions. Two interviewees noted that participation in athletics, and the normalization of continuous feedback, contributed to their ability to be vulnerable during feedback sessions. Residents commonly described being less open to critical feedback during debriefings if they lacked overall clinical confidence at the time of the event, if the resident felt that they had made a mistake or did not provide adequate leadership, or if the resident needed time for personal emotional processing. This phenomenon is generally referred to here as the need for self‐preservation. Even in residents reporting a positive mindset around feedback and interprofessional debriefing, the need for self‐preservation often superseded the perceived benefits of interprofessional feedback.

#### Interpersonal factors

The importance of relationships with the interprofessional team was a dominant theme for all residents interviewed. Longitudinal relationships allowed for mutual trust, respect, and investment in each other's development. Residents also found debriefing with the team and being vulnerable deepened new or existing professional relationships, allowing for future meaningful feedback both in and outside critical event debriefings.

Several residents noted that attending physicians had the opportunity to set the tone of a debrief that either allowed for honest exchange of feedback or more canned responses. Residents felt encouraged to be more open to critical feedback if attendings or coresidents modeled vulnerability. In addition, the resident–attending relationship preceding a critical event impacted the willingness of residents to engage in an interprofessional debriefing session. Some residents expressed feeling uncomfortable being vulnerable if they did not have an existing relationship with the attending or other members of the interprofessional team involved in the session. Unprofessional behavior between team members during the critical event or in past interactions was mentioned as a factor that discouraged a resident from initiating critical event debriefings.

When a resident felt that it was their duty as physician team leader to facilitate a debriefing for team learning and emotional processing after an event, they were more willing to engage deeply in the session and take risks for the betterment of the team. Not all residents identified with this role.

#### Institutional factors

Clinical demands and the perceived lack of time were cited most frequently as a barrier to team debriefing. Residents were hesitant to both initiate and engage in a debriefing session if they sensed reluctance from the team. If the resident sensed reluctance, either they would not initiate or they would rush through the process, limiting opportunities for meaningful feedback. A lack of private and easily accessible space was another common limiting factor. Often the debriefings were held in the room with a deceased patient. Some residents felt this interfered with their sense of safety and openness.

Standardizing the process and providing departmentwide education on the importance of debriefing helped. One resident noted that the debriefing guide's introductory script that reinforces that it is a safe space for feedback and learning was specifically helpful.

An institutional culture supportive of learning with multiple interprofessional learners allowed for vulnerability and openness to feedback. Residents were more willing to acknowledge their role as a learner if they were accompanied by other learners. When other members of the interprofessional team were also in explicit learning roles, it changed the resident's sense of hierarchy and allowed the resident to feel more comfortable also identifying as a learner.

## DISCUSSION

In resident education, critical event debriefings can provide unique opportunities to understand and learn from complex clinical situations, process emotionally charged events, identify areas for quality improvement, and strengthen relationships among the interprofessional team; however, these conversations can also threaten a resident team leader's reputation and credibility.^14^ In the high‐stakes field of medicine, revealing imperfection and weaknesses can be daunting, especially among learners.^15^ The pressure for a resident to appear competent and hide vulnerabilities impedes learning and can cause significant mental stress.^15^ Throughout residency training, residents continually work to build their credibility within the interprofessional team and with their attending physician supervisors while simultaneously growing their own fund of knowledge. It is critical to understand the success factors and limitations of psychological safety in debriefings to capitalize on the potential learning conversations or, alternatively, not cause the resident undue stress. Interviews with resident team leaders who participated in critical event debriefings show a strong tendency among residents to shield themselves from negative feedback, even at the expense of learning and personal growth, in learning environments they perceive to be unsafe. To enable to residents and the interprofessional team to gain the most from these learning conversations, it is critical that department leaders and medical educators tend to the complex array of intrapersonal, interpersonal, and institutional factors that can affect resident psychological safety when establishing or maintaining a critical event debriefing program in a training environment. While it may be impossible to predict when a resident will need to prioritize self‐preservation over learning conversations, we can work toward a greater understanding of these competing interests and develop tools to normalize, if not overcome, this tension.

Our results support having a departmentwide agreement to support an interprofessional debriefing program and providing scripted instructions for the team leader, both of which are considered best practices.^12^, ^17^ Standardizing the program, providing time and space when feasible, and encouraging participation from all members of the team will in and of itself will help develop relationships and enable open communication during future critical events. Allowing space for validating statements, and even including this in the formal script, can increase confidence, familiarity with feedback, and interprofessional bonds. This can be of particular value to junior residents as they establish relationships and gain clinical confidence. The department can further support interprofessional education and team building through other activities such as interprofessional didactic conferences, simulation, and community engagement; however, critical event debriefing provides a unique in situ experience that does not require additional outside resources or time.

Our study showed that embracing the presence of interprofessional learners can increase a resident's comfort with interprofessional feedback. Residents are part of a complex hierarchy in interprofessional teams—they are the nominal team leader but are often the novice in the room and lack the experience of the nurses and other staff on the team. When residents are either forced to or opt to defer team leadership or decision making to more experienced team members, they expose their weaknesses and vulnerabilities. Van Schaik et al.^18^ raise the possibility that education and transparency on this complex hierarchy may improve teamwork. By emphasizing the goal of interprofessional team learning and interprofessional feedback to the resident team leaner, educators can frame the conversation to best capitalize on the opportunity.

Our study further demonstrated the importance of attending partnership. Molloy and Bearman^16^ propose that by modeling intellectual candor, described as “the verbalization of thinking with respect to a genuinely complex problem or situation … without a demand for perfection,” educators can help learners take intellectual risks and embrace the tension between credibility and vulnerability. Educators can model candid learning conversations that embrace fallibility to set the tone for the learner by openly discussing uncertainty they experienced in the case, decisions they may reconsider, or prior difficult related experiences. Educators can also explicitly check in with the residents on their ability to receive feedback in the moment.

The learning conversations that take place during critical event debriefings are high‐stakes and complex scenarios for the resident physician team leader. As educators, we should strive to cultivate a safe learning environment for the resident physician team leaders to capitalize on these learning conversations and, at the very least, not cause the resident psychological distress. Further research should be done to understand how these specific interventions support psychological safety in this dynamic clinical learning conversation.

## LIMITATIONS

This study has several factors that limit the generalizability of our findings. This is a single‐center study with a limited sample size that only examines residents who have completed a critical event debriefing session in the ED. This study does not capture the perspective of residents who have not had the opportunity or have chosen not to lead a session. Further research with a larger sample size that includes residents who choose not to participate would add to the findings. Interviews were conducted by a faculty member and champion of the debriefing program and not a neutral party which may have altered participants responses.

This study also did not explore the role of age, gender, ethnicity, sexual orientation, or other personal identifying characteristics on psychological safety. The tension of credibility and vulnerability can vary depending on personal identifying characteristics. Further research that addresses these in the feedback experience is critical to understanding and improving the learning environment.

## CONCLUSIONS

In summary, this study suggests that cultivating a safe learning environment for residents in critical event debriefing involves the following key elements: (1) allowing space for validating statements; (2) supporting strong interprofessional relationships; (3) providing structured opportunities for interprofessional learning; (4) encouraging attendings to model vulnerability and set the tone for honest, specific feedback; (5) standardizing the process of debriefing; (6) rejecting unprofessional behavior; and (7) creating the time and space for the process in the workplace. Educators can address these intrapersonal, interpersonal, and institutional factors when establishing and maintaining a critical event debriefing program to capitalize on learning conversations for resident physicians.

## AUTHOR CONTRIBUTIONS

Lily Hitchner, Stacy Sawtelle Vohra—study concept and design. Lily Hitchner—acquisition of data. Lily Hitchner, Stacy Sawtelle Vohra—analysis and interpretation of the data. Lily Hitchner, Mackensie Yore, Charney Burk, Jessica Mason, Stacy Sawtelle Vohra—drafting of the manuscript, critical revision of the manuscript for important intellectual content. Lily Hitchner—acquisition of funding.

## CONFLICT OF INTEREST STATEMENT

The authors declare no conflicts of interest.

## Supporting information


Appendix A

Appendix B
