# Supplementary material for: The resident experience with psychological safety during interprofessional critical event debriefings
Source: AEM Educ Train. 2023 Apr 1;7(2):e10864. doi: 10.1002/aet2.10864 (PMC10066498; doi:10.1002/aet2.10864)
Supplement: Supplementary file 1 — Appendix A [file AET2-7-e10864-s002.docx]

**Appendix A- Semi-Structured Interview Guide**

1. Tell me about your experience with receiving feedback during debriefing sessions.
2. Describe how you feel during debriefing sessions. Do you feel like you are “psychologically safe” during these sessions, meaning, can you be vulnerable and take risks when seeking feedback without fear of negative consequences?
3. What do you think factors into this?
   1. Level of training
   2. Relationships with the team
      1. Attending physician
      2. Other learners
   3. Case outcome
   4. Clinical confidence
   5. Physical space
   6. Structure of the debriefing session
   7. Other factors
