# Supplementary figures and images for: The resident experience with psychological safety during interprofessional critical event debriefings
Source: AEM Educ Train. 2023 Apr 1;7(2):e10864. doi: 10.1002/aet2.10864 (PMC10066498; doi:10.1002/aet2.10864)

**Appendix B- Critical Event Debriefing Form**


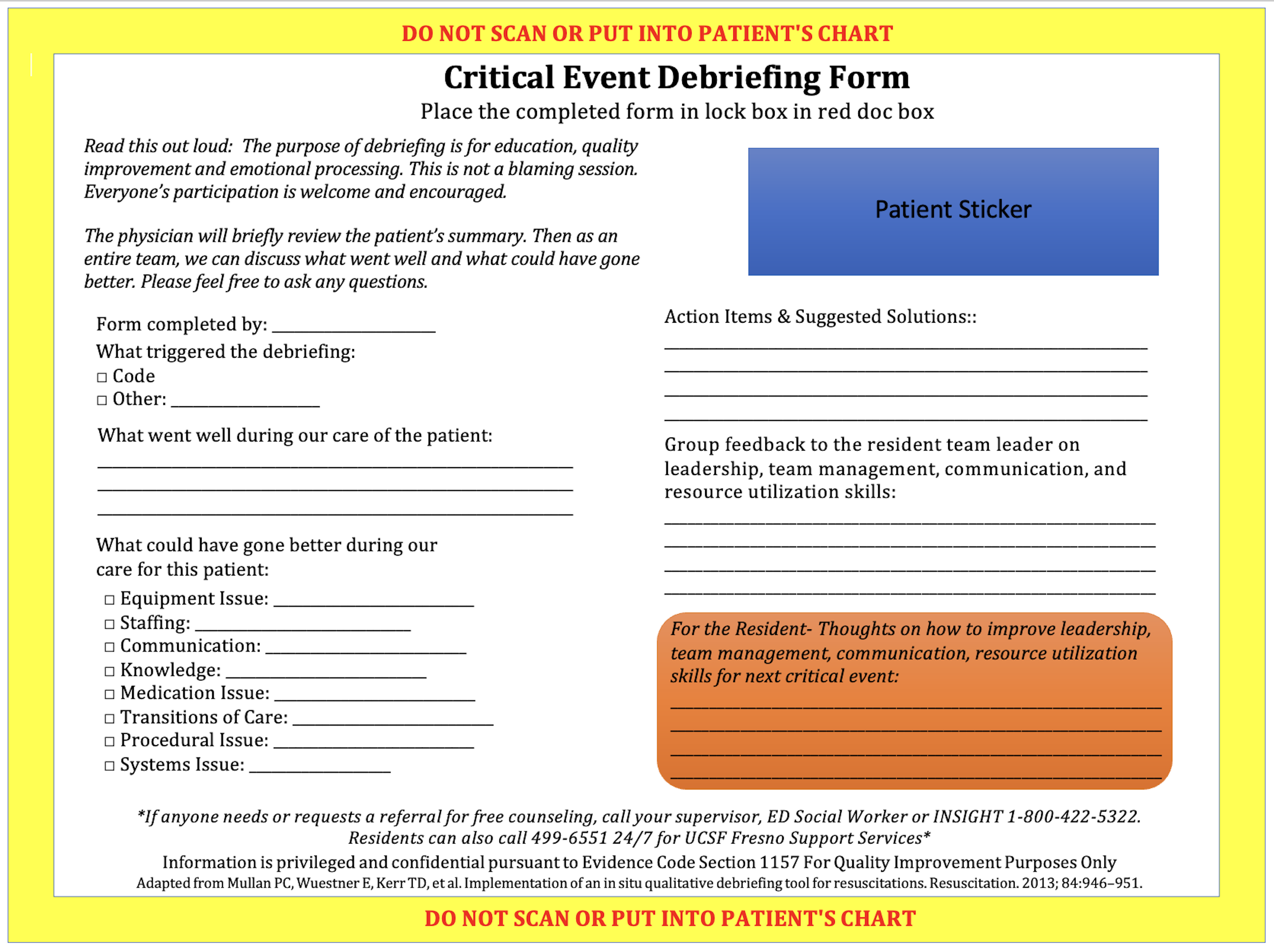

Supplement: Supplementary file 2 — Appendix B [file AET2-7-e10864-s001.docx]
